# 

**Published:** 1892-08-20

**Authors:** 


**Notices of Births, Deaths, and Marriages,** are inserted in this column at a charge of 2s. 6d. for an announcement not exceeding four lines.

### NOTICE TO CORRESPONDENTS.

All MS., Letters, Books for Review, and other matters intended for the Editor should be addressed The Editor, The Lodge, Porchester Square, London, W.

The Editor cannot undertake to return rejected M.S., even when accompanied by stamped directed envelope.

**Notice to Advertisers and Subscribers.**

All Advertisements, Orders for Copies of The Hospital, and Business Communications should be addressed to The Publisher, *not to the Editor*, The Hospital, 140, Strand, W.C.

The Cost of Subscription, post free, is as follows:—Per week, 2½d.; per quarter, 2s. 6d.; per half-year, 5s.; per annum, 8s. 6d. Foreign:—Per annum, 10s. 8d.; payable, in all cases, in advance.

**"Burdett's Hospital Annual," 1891—1892.**

Third year of publication. 600 pp. Boards, gilt lettering. A most complete guide to British and Colonial Hospitals, Dispensaries, Nursing and Convalescent Institutions, and Asylums. Price 3s. 6d. Published by The Scientific Press (Limited), 140, Strand, W.C.

**NOTICE.—The Index for Vol. XI. (ending March, 1892) is on sale at the Office of this Journal, price 2½d., post free.**

### SCRAPS AND GLEANINGS.

SIR JOHN ARNOTT has given a donation of £1,000 to the hospitals in Cork.

THE Shrewsbury Hospital Saturday Fund amounted this year to £103 14s. 2d.

HOSPITAL Saturday, in aid of the Totnes Cottage Hospital, has been fixed for August 27th.

THE Royal Albert Orphan Asylum, Bagshot, has received a grant of £10 10s. from the Drapers' Company.

AT the quarterly meeting of the Radcliffe Infirmary it was decided to put off the question of the building of the new wards until after the long vacation.

THE sale of work promoted by Miss Gilfillon and the pupils of the Luton High School for girls realized £35. This sum has been paid to the Luton Cottage Hospital.

THE Commission of Leprosy that was appointed on the initiative of the Prince of Wales, has prepared its report, along with which there will be a minority report.

THE concert at St. James's Hall in support of the Goring Thomas memorial reached £1,600, which will be devoted to founding a scholarship at the Royal Academy of Music.

IT is proposed to add two new wings to the Manchester Royal Infirmary, each wing to be about 150 feet in length. Other improvements are to be made in the present buildings.

MDME. PATTI recently gave a concert at Neath in aid of the poor of the district. She was accompanied by Signor Nicolini, Signor Bonetti, Signor Tito Mattei, and Misses Marianne and Clara Eissler.

A NEW wing has been recently added to the Stockton Hospital, and it now contains 52 beds and 8 children's cots. The income for 1891 amounted to close upon £3,000, of which sum the working classes contributed two-thirds.

THE Earl and Countess Cadogan, last Friday, entertained upwards of 2,500 of the poor of Chelsea, including 1,600 children at the Crystal Palace. The holiday was given in honour of the recent wedding of their son, Viscount Chelsea.

THE great vine at Hampton Court, the largest in England, and about 125 years old, has done remarkably well this year, and bears nearly 1,200 bunches of ripe grapes. The fruit, which is to be cut in a few days, will be packed in boxes and sent to the Queen.

A COTTAGE hospital is to be erected for the town of Nuneaton and district on a site given by Messrs. L. Tomkinson and R. Stanley, situated near the Midland Railway Station. The estimated cost of the building is £2,000. The subscriptions amount to £2,3[illegible]8 18s.

THE late Major William Vaughan Morgan, of South Kensington, bequeathed £2,000 to the London Homœopathic Hospital, a like sum to the Eastbourne Homœopathic Convalescent Home, to both of which institutions he had during his lifetime made liberal contributions.

THE National Hospital for the Paralysed and Epileptic satisfactorily maintains its benevolent activity and usefulness. The new wing is found well suited to the important uses to which it is put. The receipts for the past year were £15,539 0s. 9d., the largest since the hospital has been rebuilt.

THE total number of in-door patients admitted during the past twelve months to Noble's Isle of Man Hospital was 295, and of out-door patients 3 814 On Hospital Sunday a service was held on Douglas Head, when the Bishop preached a sermon in aid of the hospital. The offertory amounted to £30 8s. 6½d.

THE first annual report of the Fleetwood Cottage Hospital gives good proof of the usefulness of the institution. The number of in-patients treated was 28 and of out-patients 36. The Committee announce a balance in hand of £719 19s. 3d. towards the new building fund, £500 of which was given by Mrs. Fielden, £100 by Mr. J. A. Fielden, and £300 by Mr. H. C. Smith.

MR. WILLIAM SMALLWOOD, needle manufacturer, of Redditch, has left the sum of £15,000 for a cottage hospital to be built in his native town. Mr. Smallwood has also left £3,000 for the erection of almshouses, and these, with other benefactions, bring up the amount devoted to charitable and religious purposes to £26,000, of which the larger amount benefits Redditch.

AT a meeting of the Islington Guardians the chairman (Mr. J. S. Furlong) stated that a new infirmary was urgently needed for the parish in Tollington Park. Some discussion ensued, which resulted in a postponement of the further consideration of the question until the committee appointed by the opponents of the scheme could see the plans and lay their views before the board.

AT a meeting of the Ladies' Committee of the Hull Hospital Saturday Fund, it was stated that, out of the £535 12s. 4d. collected, £387 2s. 4d. had been apportioned to the Hull Royal Infirmary, £51 13s. 9d. to the Hull Victoria Hospital for Sick Children, £49 14s. 7d. to the Hull and Sculcoates Dispensary, £10 3s. 7d. to the Hull Homœopathic Dipensary, and £8 5s. 9d. to the Hull Orthopædic Hospital, a total of £507. The expenses had been £28 4s., leaving a balance of 8s. 4d.

THE total number of patients in the Cork District Asylum during the year 1891 was 48 in excess of any previous year. The number under treatment is stated to have been 1,305, namely, 654 males and 651 females, a very curious approximation of the sexes, the rule being more females than males. The inspector of lunatics on visiting the asylum expressed regret that in so large and unfortunately overcrowded an asylum no structural provision had been made for the complete isolation of patients suffering from acute infectious diseases.

A SAD fatality happened to a boating party at Matlock, comprising Mr. M'Kee, Miss Wilkins, of Farnsfield, Mr. Parkinson, of Worcester, a youth named Harry Marriott, and two boys named Stendall and Brooks. During the cruise Mr. Parkinson lost his oar, and the boat was drawn into the mill "goit" which supplies the motive-power to Sir R. Arkwright's mills. The whole of the party were thrown into the water. The two boys were picked out below the shuttle, but Marriott was crushed to death. Mr. M'Kee was rescued in an exhausted condition, while Miss Wilkins and Mr. Parkinson escaped with a drenching.

### THE STUDENT OF MEDICINE.

[All communications for this Supplement should be addressed to THE EDITOR of THE HOSPITAL, at 140, Strand, with the words, "Students' Column," written in the left hand top corner of the envelope.]

### APPOINTMENTS.

Bailey, J. Harold, M.B., Ch.B.Vict., appointed House-Surgeon to the Wirrall Children's Hospital, Birkenhead. Blakeman, Charles J., M.R.C.S., L.R.C.P.Lond., appointed Resident Medical Officer to the City Hospital South, Grafton Street, Liverpool. Boyd, James Paton, M.B., C.M.Glas., appointed Assistant-Physician to the Glasgow Royal Infirmary. Bryan, Frederick, M.B., M.R.C.S., L.S.A., appointed Senior Assistant Medical Officer to the London County Asylum, Colney Hatch. Burnett, John R., M.B., C.M.Edin., M.R.C.S., L.R.C.P., appointed House Surgeon to the East London Hospital for Children. Corner, F. M.R.C.S., L.R.C.P.Lond., appointed Medical Superintendent at the temporary Infirmary at Plaistow of the Parish of Kensington. Craig, J. W., M.B., C.M., appointed Clinical Assistant to Fife and Kinross District Asylum, Cupar. Curwen, Eliot, M.B., B.C.Camb., M.R.C.S., L.R.C.P., appointed House Surgeon to the London Hospital. Figgis, S. Bradley, M.B., C.M.Edin., appointed House Surgeon to the Deaconesses' Hospital, Tottenham. Nunes, Herbert Fitz Stephen, M.R.C.S.Eng., L.R.C.P.Lond., L.S.A.Lond., appointed House Surgeon to the Lying-in Institution and Hospital for Women, Brighton. Perkins, H. A., M.D.Edin., M.R.C.S., appointed Honorary Medical Officer to the Totnes Cottage Hospital. Simmens, Harold, M.R.C.S.Eng., L.R.C.P.Lond., L.S.A., appointed Assistant Medical to the Fulham Infirmary, Hammersmith. Smith, Ernest N., jun., appointed Assistant Medical Officer at the Infirmary of the Fulham Union. Smith, K. R., M.D.Lond., B.Sc., M.R.C.S., appointed Honorary Medical Officer to the Totnes Cottage Hospital. Sugden, H. C., M.R.C.S., L.R.C.P.Lond., L.S.A., appointed Senior House Surgeon to the Bury Dispensary Hospital, Bury. Walker, Philip C., M.A., M.B., B.Ch., B.A.O.Dub., appointed Resident Medical Officer to the City Hospital, Parkhill, Liverpool. Williams, E. R., M.R.C.S., L.R.C.P.Lond., appointed Surgeon to Her Majesty's Prison, Carmarthen. E. Valentine Gibson, M.D. Edin., appointed Superintendent and Resident Medical Officer, Victoria Infirmary, Glasgow.

### VACANCIES.

The following vacancies are announced this week, the amount of salary in each case being given after the name of the institution:

### Resident Medical Officers.

City of London Hospital for Diseases of the Chest, Victoria Park, E., House-Physician for six months, commencing October 1st.

County Asylum, Lancaster, Assistant Medical Officer—Salary, £100.

Devonshire Hospital, Buxton, House-Surgeon—Salary £100 per annum, with board, &c.

General Hospital, Birmingham, Assistant House Surgeon. Appointment for six months, with board, &c.

Kent and Canterbury Hospital, House Surgeon—Salary, £90 first year, with board, rising to £100 second year.

Kent County Luna ic Asylum, Barming Heath, Senior Assistant Medical Officer—Salary, £250 per annum, increasing two annual instalments of £25 each to £300.

London Temperance Hospital, Hampstead Road, N.W., House-Surgeon for six months—Salary at the rate of 50 guineas per annum.

London Temperance Hospital, Hampstead Road, N.W., Junior House-Surgeon for six months.

Manchester Royal Infirmary, Resident Medical Officer, doubly qualified —Salary, £150 per annum, with board, &c.

Nottingham Borough Asylum, Second Assistant Medical Officer—Salary, £100 per annum, with board, &c.

Suffolk General Hospital, Bury St. Edmunds, House Surgeon—Salary, £100 per annum.

### Non-Resident Medical Officers.

Kent County Lunatic Asylum, Barming Heath, Second Assistant Medical Officer—Salary, £180.

### The Hospital Advertiser.

### THE STUDENT OF MEDICINE—(*continued*).

London County Council, Assistant Medical Officer of Health, not less than 26 or more than 40 years of age—Salary, £300 per annum rising by annual increments of £50 until it reaches £600 a year.

Manchester Hospital for Consumption and Diseases of the Chest and Throat, Honorary Assistant Medical Officer; doubly qualified.

### PASS LISTS.

#### University of London.

INTERMEDIATE EXAMINATION IN MEDICINE, JULY, 1892.

ENTIRE EXAMINATION.

FIRST DIVISION.—A. Armer, Guy's and St. Bartholomew's Hospitals; Dorothea Caine, London School of Medicine for Women; T. V. Cunliffe, Owens College; P. J. Edmunds, B.Sc., University College; J. Ganner, Queen's College, Birmingham; S. Gillies, St. Bartholomew's Hospital; J. P. Hall, Owens College; H. G. Lawrence, St. Mary's Hospital; P. McDougall, B.Sc., Owens College; F. C. Sprawson, King's College; E. C. Taylor, Guy's Hospital.

SECOND DIVISION.—H. R. Andrews, London Hospital; E. Louise C. Appel, B.Sc., London School of Medicine for Women; J. Ashton, St. Mary's Hospital; G. F. Bergin, Bristol Medical School; J. S. Boden, King's College; J. J. Coleman, Guy's Hospital; H. W. Collier, Guy's Hospital; P. R. Cooper, B.Sc., Owens College and Manchester Royal Infirmary; F. G. Crookshank, University College; T. V. Crosby, University College; A. H. P. Dawnay, University College; J. C. Edgar, Owens College; D. E. Evans, St. Mary's Hospital; G. G. Genge, St. Thomas's Hospital; H. T. Gillett, St. Bartholomew's Hospital; T. Gregory, Owens College; A. G. Gullan, University College, Liverpool; J. C. Hibbert, University College; A. L. Home, St. Thomas's Hospital; J. H. Horton, Guy's Hospital; R. L. Jones, University College; J. P. Kitson, University College; W. D. Knocker, St. Thomas's Hospital; T. P. Legg, St. Bartholomew's Hospital; C. H. Melland, Owens College and Manchester Royal Infirmary; D. Morrison, University College; E. E. Murray, University College; M. J. O'Flanagan, B.Sc., Owens College; B. A. Richmond, Guy's Hospital; F. W. Robertson, St. Bartholomew's Hospital; R. H. Steen, Queen's College, Belfast; Helen Swatman, London School of Medicine for Women; P. N. Vellacott, Guy's Hospital; C. F. Wakefield, Guy's Hospital; G. H. C. Way, University College.

EXCLUDING PHYSIOLOGY.

SECOND DIVISION.—J. N. Brown, University College; J. S. Chater, St. Bartholomew's Hospital; G. W. Gostling, University College; M. L. G. Hallwright, Queen's College, Birmingham; G. R. Harcourt, St. Thomas's Hospital and King's College; A. Hunnard, University College, S. P. James, St. Mary's Hospital; Amelia Maitland Le Pelley, London School of Medicine for Women; W. M. Price, Guy's Hospital; A. E. Reynolds, University College; S. E. Shoppee, University College; W. Smith, Bristol Medical School; J. E. Waite, University College; A. Young, Sheffield Medical School and University College.

PHYSIOLOGY ONLY.

FIRST DIVISION.—W. Branson, Sheffield Medical School and University College; C. R. Colley, Guy's Hospital; E. F. H. Hardenberg, Guy's Hospital.

SECOND DIVISION.—R. Bebb, London Hospital; D. A. Chaning-Pearce, Guy's Hospital; S. F. Gibbs, St. Bartholomew's Hospital; T. C. Halliwell, St. Thomas's Hospital; H. W. Hart, Westminster and Guy's Hospitals and King's College; N. Instone, Guy's Hospital; J. Nicholson, The Yorkshire College; H. S. Revell, University College; W. H. Stoddart, University College.

EXAMINATION FOR HONOURS.

ANATOMY.—First class: F. J. Steward (gold medal), Guy's Hospital. Second Class: L. J. Miskin, St. Thomas's Hospital. Third Class: R. Corfe, St. Mary's Hospital; J. S. Bolton, B.Sc., University College; G. B. Hunt, University College; G. H. Sowry, St. Bartholomew's Hospital.

PHYSIOLOGY AND HISTOLOGY.—First Class: F. Fraser (Exhibition and Gold Medal), St. Bartholomew's Hospital; F. J. Steward (Gold Medal), Guy's Hospital; A. R. Cook, B.Sc., University of Cambridge. Second Class: H. Nolan, Guy's Hospital; W. S. Handley, Guy's Hospital; A. Salter, Guy's Hospital: E. J. Toye, St. Bartholomew's Hospital. Third Class: J. S. Bolton, University College; A. Bousfield, B.Sc., King's College; B. L. Abrahams, B.Sc., University College; H. J. Scharlieb, University College.

ORGANIC CHEMISTRY.—First Class: F. J. Steward (Exhibition and Gold Medal), Guy's Hospital; A. Bousfield, King's College; H. Nolan, Guy's Hospital. Third Class: W. S. Handley, Guy's Hospital; J. S. Bolton, University College; E. J. Toye, St. Bartholomew's Hospital.

MATERIA MEDICA AND PHARMACEUTICAL CHEMISTRY.—First Class: H. Nolan (Exhibition and Gold Medal), Guy's Hospital; R. S. Hardman, Owen's College and Manchester Royal Infirmary. Second Class: J. S. Bolton, University College; S. W. Brook, Owen's College. Third Class: E. J. Toye, St. Bartholomew's Hospital; R. J. Warrington, Owen's College; R. H. Norman, Westminster Hospital; F. Fraser, St. Bartholomew's Hospital; T. A. Starkey, University College; J. Currie, St. Bartholomew's Hospital.

